# Emerging role of exosome signalling in maintaining cancer stem cell dynamic equilibrium

**DOI:** 10.1111/jcmm.13676

**Published:** 2018-05-25

**Authors:** Zhen Sun, Li Wang, Lihua Dong, Xiujie Wang

**Affiliations:** ^1^ Laboratory of Experimental Oncology State Key Laboratory of Biotherapy/Collaborative Innovation Center for Biotherapy West China Hospital West China Clinical Medical School Sichuan University Chengdu China; ^2^ Laboratory of Lung Cancer, Lung Cancer Center West China Hospital West China Clinical Medical School Sichuan University Chengdu China; ^3^ Human Anatomy Department School of Preclinical and Forensic Medcine Sichuan University Chengdu China

**Keywords:** cancer cells, cancer stem cells, cell‐cell communication, dedifferentiation, dynamic equilibrium, exosomes

## Abstract

Cancer stem cells (CSCs) are a small subset of heterogeneous cells existed in tumour tissues or cancer cell lines with self‐renewal and differentiation potentials. CSCs were considered to be responsible for the failure of conventional therapy and tumour recurrence. However, CSCs are not a static cell population, CSCs and non‐CSCs are maintained in dynamic interconversion state by their self‐differentiation and dedifferentiation. Therefore, targeting CSCs for cancer therapy is still not enough,exploring the mechanism of dynamic interconversion between CSCs and non‐CSCs and blocking the interconversion seems to be imperative. Exosomes are 30‐100 nm size in diameter extracellular vesicles (EVs) secreted by multiple living cells into the extracellular space. They contain cell‐state‐specific bioactive materials, including DNA, mRNA, ncRNA, proteins, lipids, etc. with their specific surface markers, such as, CD63, CD81, Alix, Tsg101, etc. Exosomes have been considered as information carriers in cell communication between cancer cells and non‐cancer cells, which affect gene expressions and cellular signalling pathways of recipient cells by delivering their contents. Now that exosomes acted as information carriers, whether they played role in maintaining dynamic equilibrium state between CSCs and non‐CSCs and their mechanism of activity are unknown. This review summarized the current research advance of exosomes’ role in maintaining CSC dynamic interconversion state and their possible mechanism of action, which will provide a better understanding the contribution of exosomes to dedifferentiation and stemness acquisition of non‐CSCs, and highlight that exosomes might be taken as the attractive target approaches for cancer therapeutics.

## 1 INTRODUCTION

Cancer stem cells (CSCs), also named as cancer‐initiating cells (CICs), are a small subset of heterogeneous cells existed in tumour tissues or cancer cell lines.^1^ Recently, more and more basic and preclinical experimental investigations suggested that CSCs could be isolated from several types of cancers, including leukaemia^2^, ^3^, ^4^ and solid tumours.^5^, ^6^, ^7^, ^8^, ^9^ CSCs possess unlimited self‐renewal and multilineage differentiation potentials, and played very important roles in tumour initiation, recurrence, metastasis and therapeutic resistance.^10^, ^11^, ^12^ Due to their properties, CSCs were considered to be responsible for the failure of traditional (surgical operation, radiotherapy and/or chemotherapy) and target therapy as well as tumour relapse. CSCs can be isolated and characterized from tumour tissues and cell lines based on their surface markers (such as, CD133, CD24, CD90, ALDH1, etc.) and Hoechst 33342 exclusion by flow cytometry, and selectively cultured with SFM.^13^, ^14^, ^15^ CSCs have their own specific surface markers and signalling pathways, which might offer an optimistic opportunity to target eliminating CSCs and/or induce them differentiation for preclinical anti‐cancer therapy. However, recent studies indicated that CSCs are not a static cell population with the capacity of initiating tumour through asymmetric cell division, but a cell population in highly dynamic equilibrium state, which could be maintained through the dedifferentiation of matured cancer cells.^16^, ^17^, ^18^, ^19^ Until now, the CSC plasticity or dedifferentiation of matured cancer cells (or non‐cancer stem cells, non‐CSCs) with acquisition of stem‐like properties was reported in several cancers.^20^, ^21^, ^22^, ^23^

Taken together, all the findings mentioned above suggest that CSCs and non‐CSCs are not in a motionless but in a dynamic equilibrium state: CSCs differentiate into non‐CSCs under some circumstances, and non‐CSCs could dedifferentiate into CSCs. Therefore, targeting CSCs seems to be not enough, and blocking the process of non‐CSC dedifferentiation to disturb the dynamic equilibrium between CSCs and non‐CSCs appears to be particularly important for cancer therapeutics. However, the cellular and molecular mechanisms of interconversion between differentiated non‐CSCs and CSCs are unclear. More recently, emerging evidence revealed that the molecular cross‐talking between CSCs and non‐CSCs in tumour microenvironment plays a critical role in this process. The intercellular communication between tumour cells and other cells is accomplished via cell‐cell interactions. Tumour cell released growth factors, chemokines, proteins, mRNAs, microRNAs, etc., are carried and transferred by carriers. Exosomes may serve as important molecular information carriers to communicate with CSCs, non‐CSCs and other cells in tumour microenvironment.^24^

Exosomes, nanovesicles originated from the endosome, are 30‐100 nm EVs released by all types of cells.^25^ They modulate intercellular communication by transfer their molecular contents between different types of cells.^26^ Cancer cell released exosomes that play a crucial role in the intracellular communications involved in cancer progress.^27^These tumour‐cell‐derived exosomes are found in all body fluids, upon contact with target cells, they can alter phenotypic and functional attributes of recipients, reprogramming them into active contributors to tumour growth, metastasis and immunosuppression.^28^, ^29^, ^30^ However, the role of exosomes in the reciprocal conversion between non‐CSCs and CSCs was rarely investigated. As information carriers between cells, whether exosomes regulate non‐CSC dedifferentiation in CSC dynamic equilibrium, and targeting exosome signalling could attenuate the production of CSCs and finally eradicate cancers is worthy of serious study.

## 2 THE CSC PLASTICITY AND TUMOUR MICROENVIRONMENT

**Figure 1:**
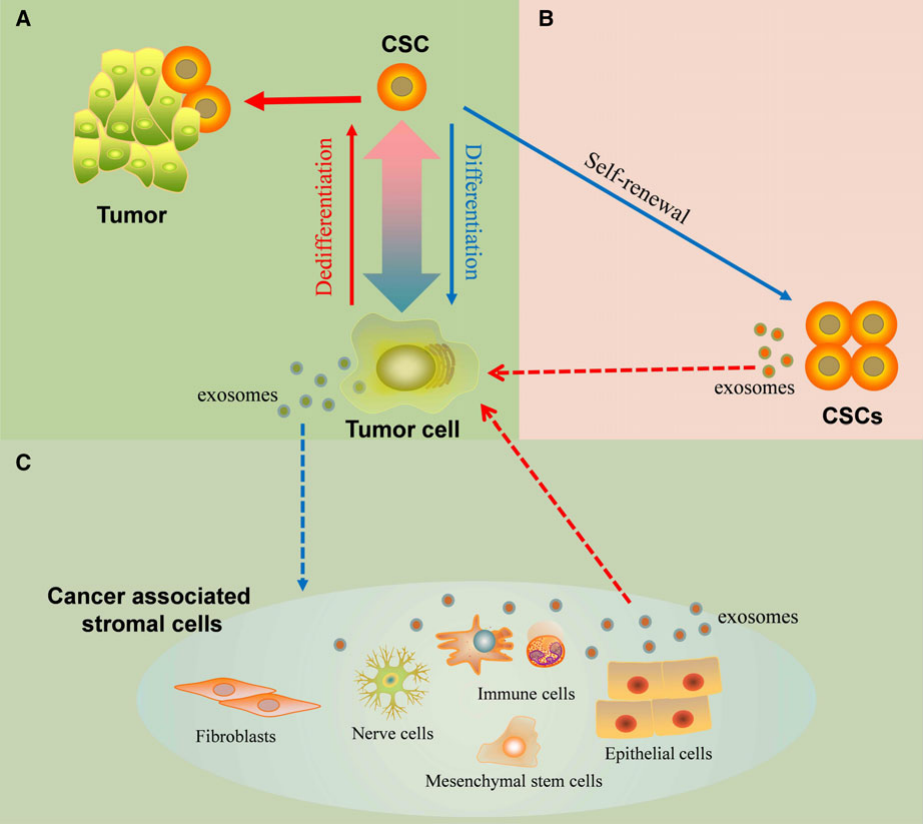
The role of exosomes in maintaining cancer stem cell dynamic equilibrium. A, The dynamic equilibrium between CSCs and non‐CSCs: CSCs differentiated into cancer cells, and cancer cells dedifferentiated into CSCs. B, C, The role of exosomes in the reciprocal conversion between non‐CSCs and CSCs: CSCs‐derived exosomes may induce the dedifferentiation of cancer cells to acquire stemness phenotype through transfer their stemness‐related molecules; cancer cell‐derived exosomes may also affect the surrounding cells within tumour microenvironment as well as these non‐tumour cells could promote tumour initiation and progression by releasing exosomes. Dotted line means the exosome‐mediated cell‐cell communication

Emerging evidence have been found that non‐CSCs could be reprogrammed and transform into CSC‐like cells, which indicated that CSCs and non‐CSCs were in a dynamic equilibrium state (Figure 1A), and the molecular crosstalk between cancer cells and CSCs in tumour microenvironment seems to play an important role in maintaining of this dynamic equilibrium.

Tumour microenvironment consisted of a variety of different cell populations including cancer cells, CSCs, mesenchymal stem cells (MSCs), endothelial cells, fibroblasts, bone marrow‐derived cells (BMDC), etc.,^31^ and recent studies demonstrated that cells within tumour microenvironment can regulate cancer cell stemness phenotype, promote tumour cell invasion and metastasis, induce angiogenesis, and immune cell recruitment by secreting various bioactive factors, these factors were also involved with the conversion of non‐CSCs into CSCs. For example, high‐mobility group box 1 (HMGB1) released by cancer‐associated fibroblast (CAFs) was found to enhance stemness and tumorigenicity of breast cancer cells through HMGB1/TLR4 signalling pathway^32^; CAFs‐derived HGF could activate FRA1/HEY1 signalling pathway to endow hepatocellular carcinoma cells with stemness phenotype.^33^ Except for these transcription factors, CAFs‐derived microRNA‐149 also acted as an important player in CAFs‐mediated stemness phenotypic acquirement of gastric cancer cells.^34^, ^35^ Tumour‐associated macrophages (TAMs) and myeloid‐derived suppressor cells (MDSCs) are the main cell types derived from bone marrow in tumour microenvironment. Recent study showed that M2 TAMs‐derived prostaglandin E2 (PGE2) could endow colon cancer cells with stem‐like qualities; this effect was abolished by celecoxib, the COX‐2‐selective inhibitor, which blocks PGE2 production.^36^ Similarly, other cytokines such as EGF, TGF‐β1 and IL‐6 released by TMAs also promote transforming of non‐CSCs into CSCs.^37^, ^38^, ^39^ MDSCs played an important role in stemness phenotypic plasticity of non‐CSCs through activation of notch signalling, EMT and up‐regulation of stemness genes, such as, Nanog, Oct4 and Sox2,^40^, ^41^ previous study showed that MDSCs released IL‐6 activated stat3/notch signalling pathway in breast cancer cells and endow these cells with stem‐like properties.^42^ In addition, CSCs could also dictate the characters of their surrounding stromal cells by secreting a variety of factors to promote tumour progression. The study showed that CSCs induced the transition of fibroblasts to CAFs by secreting TGF‐β, breast CSCs can produce IL6, which attracts and activates MSCs to produce the CSC‐supportive cytokine CXCL7.^43^

Interestingly, recent studies reveal that a set of stemness and EMT‐related factors can transform non‐CSCs into CSCs. For instance, Suva et al identified that stemness related factor, Sox2 was involved in reprograming differentiated GBM cells to stem‐like glioblastoma cells. Similarly, in human colon cancer, a set of TFs such as OCT3/4, SOX2 and KLF4 could transform colon cancer cells into CSCs. In addition, EMT‐related factor, Snail was found to endow gastric carcinoma cells with CSC characteristics. In prostate cancer, Ruan et al also found that Twist could augment prostate cancer stem cell population. If these stemness and EMT‐related factors mentioned above were packaged by carriers, like exosomes, they could be protected from damage by protease or RNA enzyme existed in tumour microenvironment; when these biofunctional factors are carried to and uptaken by recipient cells, such as, non‐CSCs, the phenotypes of recipient cells would be changed. However, no evidence showed that exosomes carried the factors such as OCT3/4, SOX2, KLF4 or EMT‐related factors and transferred them among the cells.

Previous studies showed that cancer cells can release and uptake small EVs containing a subset of the membrane and cytosolic proteins, RNA and lipids within the tumour microenvironment, which leads to reprogramming of recipient cells, including stemness phenotype.^44^ Thus, it is reasonable to assume that EVs, including exosomes, might mediate the cell communication between non‐CSCs and CSCs, and play important roles in maintaining the dynamic equilibrium state of CSCs.

## 3 EXOSOME BIOLOGY

The term “exosome” was first used to describe the exfoliated micro‐vesicles ranging from 40 to 1,000 nm released by various normal and neoplastic cells with ectoenzymes activity by Trams et al in the early 1980s.^45^ In 1983, Stahl et al^46^ and Johnstone et al^47^ found that transferrin receptors associated with small 50 nm vesicles were literally jettisoned from maturing blood reticulocytes into the extracellular space. In the late 1980s, the name “exosome” was coined for small endosomal origin vesicles (30‐100 nm) that are released during reticulocyte differentiation as a consequence of the fusion of multivesicular bodies (MVBs) with plasma membrane by Rose Johnstone. Further studies revealed that exosomes contained signalling molecules for cell communication, such as proteins, RNAs, microRNA and lipids,^48^ which exert very important bio‐functions in the regulation of cellular phenotypic alterations through regulating the reprogramming genes and signalling pathways in recipient cells.^49^

## 4 EXOSOME BIOGENESIS AND CONTENT SORTING

**Figure 2:**
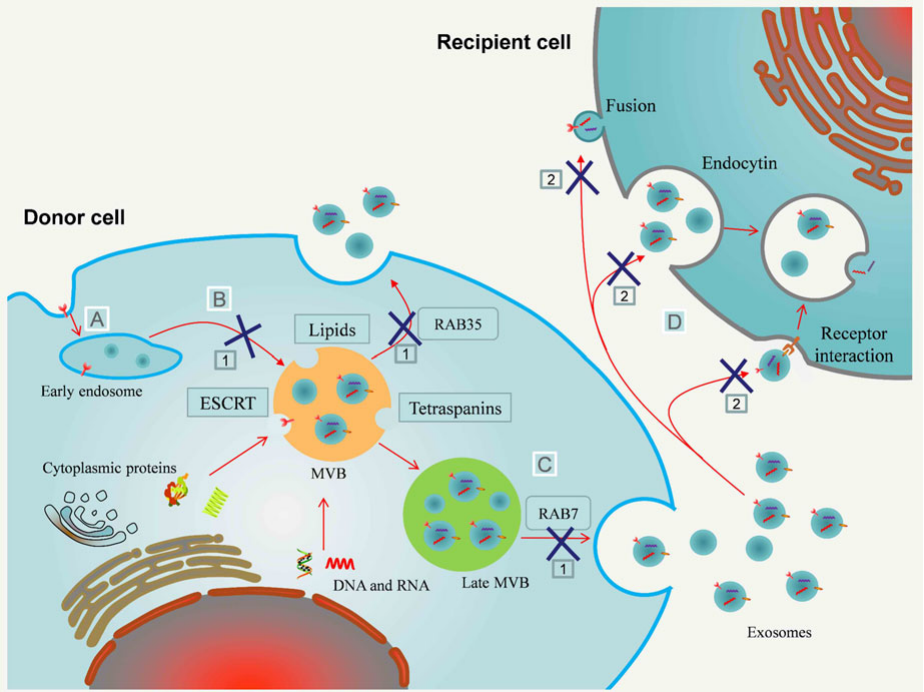
Exosome biogenesis, secretion, uptake and therapeutic targeting of exosome signalling. A, B, Exosomes are initiated from early endosomes, and formed as ILVs by budding into the early endosomal membrane through ESCRT, tetraspanins and lipid‐dependent mechanisms. C, The endosomes transform into MVBs, and then, these MVBs subsequent fuse with the plasma membrane to release the exosomes into extracellular space. D, Once released, exosomes can be internalized by neighbouring or distal cells through endocytic processes, or directly fusion with the cellular membrane. There are different possibilities to interrupt exosome‐mediated signalling: ×1 Inhibiting the process of exosome biogenesis by interfering MVB formation and/or theri release; ×2 Interrupting exosome uptake in recipient cells by blocking exosome ligands or cell surface receptors involved in exosome binding or internalization

The generation of exosomes is a very tightly regulated process governed by various cellular and molecular mechanisms. It is initiated from early endosomes (Figure 2A) and formed through the internalization of the plasma membrane. After invagination, intraluminal vesicles (ILVs) are formed by inward budding of the early endosomal membrane and accumulate in endosomes. And then the endosomes transform into multivesicular bodies (MVBs).^50^ During this process, the cytoplasmic DNA, RNA and proteins are specifically sorted into ILVs (pre‐exosomes; Figure 2B). Interestingly, evidence also confirmed that the proportion of proteins and RNAs in exosomes was different from that in the originating cells, which indicates that there are some specific mechanisms involved to control the sorting process of specific contents into exosomes. Actually, the specific sorting process of proteins cargo into exosomes is regulated by various pathways, including endosomal sorting complexes required for transport (ESCRT),^51^ tetraspanins^52^ and lipid‐dependent mechanisms.^53^ Moreover, researchers further found that a zip code in the 3′‐UTR presented in exosomal mRNAs, which may guide their sorting to exosomes.^54^ Other studies also found that a specific motif (GGAG) has been involved in the loading of specific miRNAs into exosomes through the interaction with specific chaperone proteins.^55^

## 5 EXOSOME RELEASE

Once formed, these MVBs will be mobilized to the cell periphery, and subsequently fused with the plasma membrane to release the exosomes into extracellular space.^56^, ^57^ In this process, several cellular model systems, including cytoskeleton, Rab GTPase and the fusion machinery, were found to be involved in transporting MVBs to the sites of the plasma membrane and their docking.^58^, ^59^ After docking of two different intracellular compartments, the SNARE complexes drive the fusion of MVBs with the plasma membrane, and finally exosomes were secreted into outer‐cellular milieu 50, 60 (Figure 2C). Interestingly, the secretion of exosomal microRNA (miRNA) can be regulated by the neutral sphyngomyelinase 2 (nSMase2), the release of exosomes was reduced after inhibiting the activity of nSMase2 with GW4869, and overexpression of nSMase2 increased extracellular amounts of miRNAs, hypoxia promoted exosomes release along with exosomal miRNA increase, while some drugs could inhibit exosome secretion.^61^, ^62^, ^63^ These findings indicate that the generation and secretion of exosomes are of modulation and selectivity, and whether this property could be used for cancer therapy needs deeper study.

## 6 EXOSOME UPTAKE

The elicitation of exosomal functions mediating cell‐cell communication happened after the uptake by recipient cells. The first step of uptake is the binding of exosomes to the surface of recipient cells, which is mediated by the specific receptors.^64^, ^65^ After initial binding, exosomes will be internalized by recipient cells through endocytic processes, or directly fusion with the cellular membrane and releasing their contents into the cytoplasm and finally regulate cellular pathways^66^, ^67^ (Figure 2D). In addition, a recent study showed that treatment of exosomes with proteinase K significantly reduced their uptake by cancer cells.^68^ This indicates that the uptake of exosomes by recipient cells might not be a random process, and specific molecules expressed on exosomes may serve as receptors for uptake. Therefore, blocking the binding between exosomes and recipient cell might be a promising strategy for inhibiting exosome uptake.

## 7 EMERGING ROLE OF EXOSOMES IN MAINTAINING CSC DYNAMIC EQUILIBRIUM

**Figure 3:**
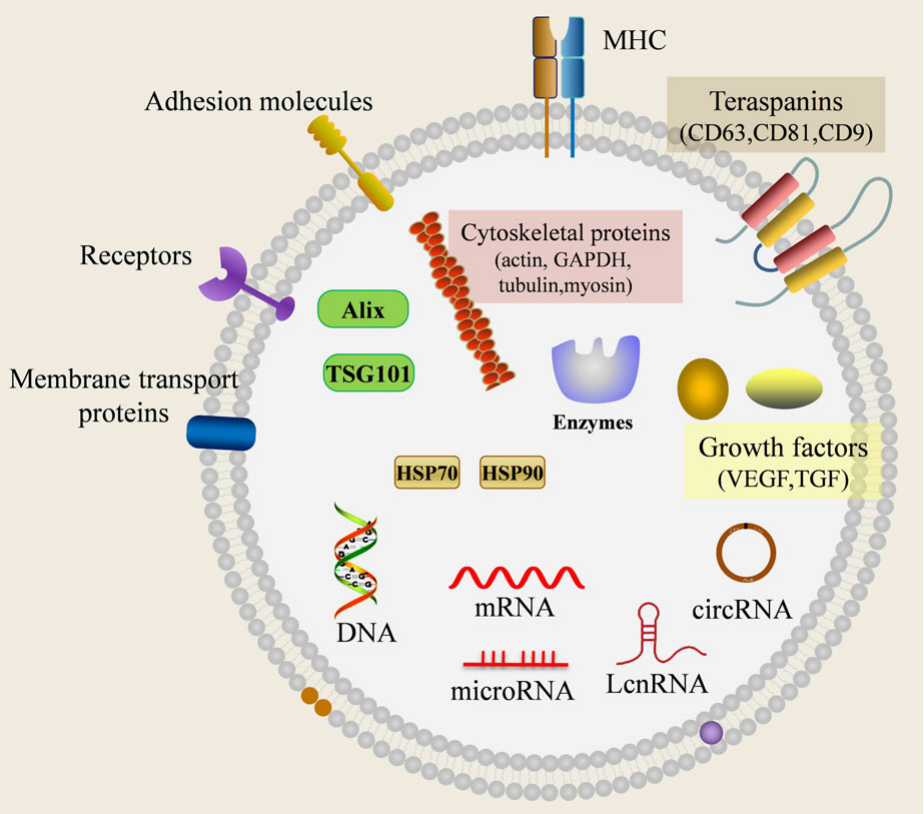
Schematic diagram and molecular composition of exosomes. Exosomes are membrane‐derived 30‐100 nm nanovesicles released by all types of cells, and contain a broad array of biologically active materials, including nucleic acids (such as mRNA, miRNA, lncRNA, circular RNAs), proteins (eg CD63, CD81, HSP70 and other origin‐specific molecules) and lipids

As mentioned above, exosomes contain a broad array of biologically active materials, including proteins, microRNAs and mRNAs, which are specifically equipped to mediate cell‐cell communication via transfer of their compositions to recipient cells^26^, ^69^, ^70^ (Figure 3). In the interconversion between CSCs and non‐CSCs, exosomes may be taken granted as important signalling information transmitter by transferring stemness‐related molecules to non‐CSCs, which lead non‐CSCs to regain stemness phenotype. On the one hand, cancer cells could recruit and alter phenotypic and functional attributes of stromal cells in tumour microenvironment by secreting exosomes (Figure 1B). On the other hand, those educated cells or CSCs transport specific molecules which are needed for tumour growth, metastasis and drug resistance into the cancer cells via exosomes to enhance tumorigenicity or stemness phenotype of cancer cells (Figure 1B, C).

It is becoming increasingly evident that exosomes derived from tumour cells impact the surrounding stromal cells. Webber et al^71^ demonstrated that exosomes produced by prostate cancer cells induced fibroblast differentiation to a myofibroblast‐like phenotype that supports angiogenesis in vitro*, and tumour growth* in vivo. Pang et al^72^ showed that pancreatic cancer cells convert normal fibroblasts to cancer‐associated fibroblast‐like cells by means of secreted EV containing miR‐155.

Moreover, exosomes derived from stromal cells within tumour microenvironment contributed to the conversion of non‐CSCs into CSCs. For example, carcinoma‐associated fibroblasts (CAFs) derived exosomes were found to endow colorectal cells with stemness phenotype, including sphere‐formation and tumorigenic capability via activation of Wnt signalling pathway, and finally increasing the percentage of CSCs.^73^ Donnarumma et al^74^ identified three miRNAs (miR‐21, ‐378e and ‐143) enriched in exosomes derived from CAFs, which could significantly increase the ability to form mammospheres, and promote the stemness and EMT phenotype of breast cancer cells. Similarly, human mesenchymal stem cell (MSC)‐derived exosomes could activate Wnt signalling pathway in recipient breast cancer cells and then promote cellular proliferative ability.^75^ In addition, Boelens et al^76^ proved that exosomes released from stromal cells can activate STAT1‐dependent antiviral signalling and NOTCH3 pathways in breast cancer cells and regulate stroma‐mediated expansion of therapy‐resistant cells.

Furthermore, exosomes derived from aggressive cancer cells, especially the CSCs could transport oncogenic factors to recipient cells within tumour microenvironment to induce tumour aggression and progression. For example, colorectal cancer‐initiating cells (CoCIC)‐derived exosomes could transfer claudin‐7 to poorly metastatic cells, consequently, significantly enhanced migratory activity of recipient cells.^77^ Nasopharyngeal carcinoma (NPC) cell‐derived exosomes are enriched in hypoxia‐inducible factor‐1α (HIF1α), which could increase migration and invasiveness of NPC cells.^78^ Besides, chloride intracellular channel‐1 (CLIC1) was found existed in CSC‐released EVs, which could induce GBM cell proliferation in vitro and tumour growth in vivo.^79^ Moreover, tumour‐supportive miRNAs, miRNA‐21 and 34a, were reported to be abundant in a wide range of cancer cells and their released exosomes.^80^, ^81^, ^82^, ^83^ Importantly, miR‐21 has shown to promote cell proliferation and tumorigenesis^84^ as well as enhance stemness phenotype of glioblastoma cells^85^ by targeting upstream or downstream genes. In addition, miR‐200 was found to enriched in EVs from metastatic breast cancer cells which transferred to non‐metastatic cells and then promoted mesenchymal‐to‐epithelial transition of recipient cells.^86^ Challagundla et al^87^ found that neuroblastoma (NBL)‐derived exosomes could transfer miR‐21 into human monocytes, and then up‐regulate miR‐155 levels to enhance NBL drug resistance. Moreover, certain stemness and metastasis‐related mRNA were found to be enriched in breast cancer stem‐like cell‐derived exosomes that could stimulate tumour progression.^88^

Besides, several studies demonstrated that long non‐coding RNA (lncRNA) was also selectively packaged in extracellular vesicles and transported to other cells, with subsequent modulation of cellular function.^89^ LncRNAs are broadly classified as capped transcripts longer than 200 nucleotides, which could regulate chromosome remodelling, transcription, translation, and protein modification, and misregulation of lncRNA involved in cancer development and progression.^90^, ^91^ For example, exosomes derived from CSC‐like CD90+ liver cancer cells contain lncRNA H19, which could promote angiogenesis and cell‐to‐cell adhesion.^92^ Furthermore, circular RNAs (circRNAs) regulate gene expression at the transcriptional or post‐transcriptional level by acting as miRNA sponges, or binding to RNA‐associated proteins,^93^ and play potential roles in multiple disease processes, including tumorigenesis,^94^, ^95^ were found in exosomes and even double‐stranded DNA molecules^96^ exist in exosomes as well, while few study discovered their involvement in the regulation the dynamic interconversion between non‐CSCs and CSCs.

These studies mentioned above indicated that exosomes derived from aggressive cancer cells, especially the CSCs or stroma cells within tumour microenvironment could regulate cellular signalling pathways in non‐CSCs involved with stemness phenotype while the release and uptake of exosome could be controlled. From these, a comprehensive therapeutic strategy targeting exosomes to eradicate CSCs for cancer therapeutics would be more effective.

## 8 EXOSOMES AS TARGET FOR CANCER THERAPY

As described above, exosomes acted as information carriers by delivering their components to recipient cells, mediated communications between cells and resulted in phenotypic and bio‐functional reprogramming of recipient cells. Exosomes derived from CSCs might mediate the cell communication between non‐CSCs and CSCs, and maintain dynamic equilibrium state of CSCs in tumour microenvironment, by transferring stemness molecules to non‐CSCs. Therefore, targeting exosomal signalling pathways may break down this equilibrium, which might be a novel and better strategy for cancer therapeutics compared with targeting killing both CSCs and non‐CSCs.

Fortunately, recent studies revealed the possible mechanisms involved in the exosome biogenesis, release and uptake; thus, many potential strategies to interrupt exosome‐mediated signalling can be envisioned (Figure 2). For example, the ESCRT machinery is known to be involved in the formation of MVBs and ILVs, and knockdown the components of ESCRT, such as HRS,^97^ STAM1 or TSG101,^51^ could inhibit the exosome biogenesis. Besides ESCRT machinery, several lipids and lipid metabolizing enzymes were suggested to also regulate this process in some cells.^52^, ^98^ Inhibiting nSMase activity by hydrochloride hydrate (GW4869) or RNAi could reduce exosome production and prion packaging.^99^ Rab27 family is small GTPases to regulate exosome release. Knockdown Rab27 via RNAi reduced exosome release, tumour growth and the dissemination of metastatic colonies significantly,^100^ Conversely, overexpression of EPI64, a candidate GAP that is specific for Rab27, could promote exosome secretion in lung cancer cells.^101^ Exosomes could be internalized by recipient cells through endocytic processes, or directly fusion with the cellular membrane, heparan sulphate proteoglycans have been implicated in this process, and treatment with heparin significantly inhibit exosome uptake by cancer cells.^102^

Another strategy might be involved in manipulation of the exosome cargo. For example, treatment with vemurafenib, the BRAF inhibitor, significantly increased the total RNA and protein content of the released EVs and caused significant changes in the RNA profiles in the vesicular secretome of malignant melanoma cells.^103^ In addition, inhibition of ESCRT components or aSMase activity also modulates the nature and content of the vesicles.^104^

Therefore, inhibition of exosome biogenesis, release, uptake or modification of exosome cargo in tumour microenvironment may have beneficial effects on blocking the interconversion between CSCs and non‐CSCs.

## 9 EXOSOMES AS DRUG‐DELIVERY VEHICLES FOR TARGETED CANCER THERAPY

Unlike synthetic nanoparticles, the lipid bilayer membrane of exosomes can pass through the blood‐brain barrier and have low toxicity and immunogenicity.^105^ Anti‐inflammatory or chemotherapeutic drugs delivered by exosomes exhibited much stronger stability, bioavailability and effectiveness, and no toxicity compared with conventional therapy.^106^ Perhaps, exosomes have great potential for targeting CSCs. For example, in a human lung tumour xenografts model, exosomes‐delivered paclitaxel showed significantly inhibit tumour growth in vivo with remarkably lower systemic and immunologic toxicities as compared with i.v. injection of paclitaxel.^107^ Moreover, exosomes can be used as natural nanovesicles to deliver exogenous small RNAs, including siRNA and microRNAs, to target tissues and/or cells for gene therapy, the targeting effect of exosomes on cancer cells can be enhanced by modifying their surface molecules, such as, coated with CSC marker antibodies and anti‐cancer drugs. Exosomes designed to express iRGD‐Lamp2b showed highly efficient targeting potential and Dox delivery capacity to αv integrin‐positive breast cancer cells in vitro and in vivo. Besides anti‐cancer therapeutic drugs, exosomes can also deliver various tumour antigens. Thus, exosomes could present CSC‐specific antigens to T cells and activate T cells for anti‐CSC immunization.^105^ In addition, exosomes can be used as natural nanovesicles to deliver exogenous small RNAs, including siRNA and microRNAs, which target CSC‐specific signal pathways, such as Wnt, Notch, Hippo, and Hedgehog, etc. for CSC targeting therapy.

Therefore, developing more specific CSC targeting exosomes will be a promising cancer therapy strategy in the future.

## 10 CONCLUDING REMARKS

Originally, CSCs were thought to be a fixed subset of cancer cells with stem cell‐like properties existed in several types of cancers and responsible for cancer initiation, progression, metastasis, recurrence and resistant to chemoradiotherapy used conventionally, CSC presumption will have an important effect on the strategies for cancer therapy clinically. However, increasing investigations indicated that CSCs and non‐CSCs are highly dynamic cell populations, with continuous differentiating, and dedifferentiating and being replenished. This might explain why single specific anti‐cancer and anti‐cancer stem cell drug or combination is unable to kill non‐CSCs and CSCs as well as both. Recent studies suggested that exosomes mediate intercellular communication among different types of cells, regulating gene expressions and cellular signalling pathways of recipient cells by delivering their components, such as proteins, RNAs, microRNAs, lipids, etc.

Based on that exosomes‐mediated transformation between non‐CSCs and CSCs in tumour microenvironment mentioned above, targeting exosomes would be a promising strategy for cancer therapy. On the one hand, more and more molecular targets in exosome regulating non‐CSCs dedifferentiating were explored and corresponding molecular inhibitors usher in the dawn such as GW4869, heparin, etc.; on the other hand, controlling exosome biogenesis, release or uptake through regulating donor or recipient cells could also block non‐CSCs dedifferentiating. Moreover, exosomes as drug‐delivery vehicles attracted a wide spread attention; through structure modification, such as surface marker enhancing or replacing, the designed exosomes are more suitable for delivery vehicles, and possess high specificity and stability.

Taken together, exosomes existed in the tumour microenvironment, acted as information carriers, played important and essential roles in maintaining the dynamic equilibrium state between non‐CSCs and CSCs. Facing the difficulties and challenges in effectively targeting CSCs for cancer eradication, future studies will be focused on comprehensive understanding cell‐specific biogenesis, content sorting of exosomes, and exosomes‐recipient cell affinity^108^ inhibiting exosome biogenesis, blocking their release and/or uptake by non‐CSCs and other cells in tumour microenvironment, and destroying the interconversion and dynamic equilibrium state between non‐CSCs and CSCs, which may open new avenues for cancer therapeutics.

## ACKNOWLEDGEMENT

This work was supported by National Natural Science Foundation of China (Grant number 31371148).

## CONFLICT OF INTEREST

The authors declare that they have no conflicts of interest.
